# Pathophysiology of SARS-CoV-2 Infection in the Upper Respiratory Tract and Its Relation to Breath Volatile Organic Compounds

**DOI:** 10.1128/mSystems.00104-21

**Published:** 2021-07-27

**Authors:** Moria Lichtenstein, Sondra Turjerman, Jayant M. Pinto, Orna Barash, Omry Koren

**Affiliations:** a NanoScent Labs Ltd., Teradyon Industrial Park, Israel; b Azriely Faculty of Medicine, Bar-Ilan University, Safed, Israel; c Section of Otolaryngology-Head and Neck Surgery, Biological Sciences Division, The University of Chicago, Chicago, Illinois, USA; Johns Hopkins Bloomberg School of Public Health

**Keywords:** microbiome, severe acute respiratory syndrome coronavirus 2, volatile organic compounds, upper respiratory tract infections, COVID-19

## Abstract

Among the many products of metabolic processes are volatile organic compounds (VOCs). In the airways, these volatile metabolites are emitted through breathing and thus are easily sampled for analysis. Recent work has connected the functions and structure of the human microbiome with health and disease. Alteration in microbial function in this context can result in differences in metabolite composition, including that of VOCs, presenting the possibility of a new noninvasive method for clinical diagnosis. Screening methods that assess VOCs arising from changes in the airway microbiome could be highly useful in diagnosing viral upper respiratory tract infections (URTIs), e.g., COVID-19, which are highly contagious and have an enormous public health impact worldwide. A rapid noninvasive screening test for URTIs would pose major advantages in containing the disease. As early evidence shows that severe acute respiratory syndrome coronavirus 2 (SARS-CoV-2) infection alters the human microbiome (both in the gut and the respiratory tract), we propose that detection of a VOC signature of an altered nasal microbiome could be fruitful as a rapid noninvasive measure of URTI in general and of SARS-CoV-2 in particular.

## OPINION/HYPOTHESIS

Breath analysis focuses on the detection and, when possible, identification of volatile compounds in the air emitted in the human breath. Volatile organic compounds (VOCs) have been shown to be useful as biomarkers for the identification of various conditions and diseases, such as inflammatory bowel disease (1). Changes in VOC patterns may mirror metabolic perturbations in the whole body at large or in specific tissues. Recent publications have extended these links to the gut microbiome related to metabolic changes. Indeed, one practical area that shows promise is in the case of infection, where the human microbiome becomes dysbiotic (1, 2). This raises the possibility of using breath analysis to detect infections, bacterial or viral (3). Furthermore, breath collection is relatively simple (compared to that of blood or invasive swabs); thus, performing clinical diagnosis based on VOC measurement methods is widely researched (4, 5).

VOCs in humans are generated during both normal physiological processes in the body’s tissues and those related to disease (4). While largely unidentified, some pathways of certain VOCs have been found and characterized. For example, saturated hydrocarbons, such as ethane and *n*-pentane, have been found to be markers of lipid peroxidation, a metabolic pathway associated with oxidative stress and subsequent tissue damage (6). Aldehydes are also metabolites of oxidative stress and have previously shown potential as volatile biomarkers for lung cancer as well as respiratory viral infections (7, 8).

Many of the published studies regarding clinically relevant VOCs describe patterns rather than specific compounds to further the understanding that many distinct processes throughout the patient’s body contribute to the identified change in the VOC profile of the human breath. Furthermore, some of the VOCs found in breath are suspected to be blood born, thus making it harder for us to identify their origins. Single cell lines may be useful in researching cell-specific VOCs and were already used to investigate VOC emission profiles of cancerous cells, immune cells (mostly neutrophils and B cells), virus-infected cells, and bacteria. All cells produce VOCs to some extent—it is their relative abundance and response that leads to condition-specific changes in the VOC profile of exhaled breath (9).

Although not as well characterized, VOCs also play a role in human-associated microbiomes in the oral cavity, gastrointestinal tract, and respiratory tract, as the resident bacterial populations produce different volatile metabolites as part of their activity, whether physiological or pathological (4).

Disease-associated changes in the human microbiome have also been placed under intense scrutiny (10, 11). Numerous studies demonstrated significant differences in microbiome composition and function between healthy and disease states at the tissue interfaces with the environment, such as respiratory tract diseases (asthma and lung cancer) (11) and gastrointestinal illness (inflammatory bowel disease) (1, 10). Alterations in the microbiome and generated volatilome have also been identified in various infectious diseases in these sites, such as influenza (3).

Considering current knowledge regarding microbial changes due to infection and disease, as well as the consequential alteration in the volatilome, we hypothesize that changes in airway VOC patterns of COVID-19 patients result from viral-induced alterations in the upper respiratory airway microbiome, both in composition and function. The development of rapid methods for assessing these changes could provide useful clinical information.

## SARS-CoV-2 PATHOPHYSIOLOGY

Severe acute respiratory syndrome coronavirus 2 (SARS-CoV-2) is an emerging pathogen that causes coronavirus disease (COVID-19). Since it was first described, >191 million infections have occurred around the world, with more than 4.1 million reported deaths to date (https://www.worldometers.info/coronavirus/; 12). While the viral load in the upper respiratory tract is highest at the time of symptom onset, infectiousness begins several days prior, which makes screening and pandemic mitigation challenging (13).

Molecular studies of SARS-CoV-2 revealed that the virus enters target cells through a membrane protein, angiotensin-converting enzyme 2 (ACE2). Nasal goblet cells and ciliary cells express higher levels of ACE2 than other cell types in the upper respiratory epithelium. Thus, the nose is thought to be the primary site of viral replication and serves as a reservoir for further dissemination in the host and transmission to others. In the gastrointestinal tract, ACE2 was found to be an important regulator of expression of antimicrobial peptides and, subsequently, gut microbiome ecology (13).

Furthermore, up to 90% of inspiration is through the nose; thus, the sinonasal cavities are a major infection site for SARS-CoV-2. Following infection with SARS-CoV-2, there are three possible phases: (i) early infection, (ii) pulmonary phase, and (iii) hyperinflammatory phase. The initial phase involves viral replication and mild symptoms if any. In the second phase, the adaptive immune system is stimulated and respiratory symptoms appear. By the third and final phase, the immune response causes a hyperinflammatory condition, which can lead to organ failure and death (13).

In this perspective, we will focus on the mechanism of infection as well as the first phase of the disease, as these stages are relevant for symptomatic and asymptomatic identification alike and are the phases in which most human-to-human infections occur.

## TARGETING MICROBIAL CHANGES IN THE NASAL CAVITY AS A METHOD FOR EARLY SARS-CoV-2 DETECTION

Natural microbial communities can be interrupted by infections, noninfectious diseases, and environmental exposures. Here, we focus on effects related to viral infections to inform consideration of how VOCs from an altered microbiota could be exploited in COVID-19 diagnosis.

### Microbiome alterations due to viral infection.

Different individuals and populations react differently to specific viral pathogens; the timing of the immune response, the manifestation of symptoms, and even fatality may vary. Such variation may be due to age, sex, race, genetics, or underlying clinical conditions, all of which are factors known to influence microbiome composition (14).

A large body of work has shown that the human gut microbiome can modulate and even moderate many host functions, including the immune response and susceptibility to infections (10). These properties are not limited to the microbiome’s location in a specific organ, as microbiome-host interactions can be far reaching, meaning that the microbial community in the gut can influence other organs (15). Conversely, in addition to modulation of the viral infection by the microbiome, alterations in the human microbiome have also been reported following viral infection. For example, influenza decreases colonization of healthy commensal bacteria in the upper respiratory tract while increasing the presence of potentially pathogenic bacteria (15). Changes in the microbiome are not only compositional; significant changes can occur in microbial metabolism, even if no notable alteration in populations is observed (14).

The nasal microbiome may respond to a number of airway diseases, including asthma, allergic rhinitis, and respiratory infections. Similarly, microbiome composition has been shown to differentially affect airway disease progression (16). For example, Wen et al. (17) showed a distinct nasal microbiota in influenza A-infected children compared to those in their healthy peers. Contrarily, the oropharyngeal microbiota showed great stability after influenza infection, leading to the notion that the oropharyngeal microbiome is less susceptible to viral changes (17).

### Microbiome alterations in COVID-19.

Only a few studies have examined the alterations in the gut microbiome followed by SARS-CoV-2 infection. One such study found statistically significant differences in bacterial diversity between the microbiomes of COVID-19 patients, influenza patients, and healthy controls. Furthermore, specific bacterial genera serve as biomarkers for differentiation between COVID-19, influenza, and healthy microbiomes, at least in the gut (18).

Many COVID-19-related studies of the microbiome discuss its ability to modulate an individual’s susceptibility to the infection and their consequential immune response (13). Changes in the microbiome also likely occur among COVID-19 patients after infection. These changes may be qualitative, leading to an alteration in bacterial composition, or quantitative, causing a change in the abundance of each taxon, and may involve a change in the function of bacteria as well, including altered secretion of metabolites, which can be detected in blood, urine, feces, and breath.

The effect SARS-CoV-2 infection has on the upper respiratory microbiome is less known. Haiminen et al. (19) compared COVID-19 patients to healthy peers and to non-COVID-19-related pneumonia patients. After successfully differentiating patient groups by microbiome composition, they detected significant changes in bacterial metabolism pathways (19). This study suggests that, as in other health conditions, metabolically detectable changes in the microbiome occur in SARS-CoV-2 infection.

De Maio et al. profiled 40 nasopharyngeal swabs that were previously used to determine whether a subject was COVID-19-positive or negative (by reverse transcription PCR [RT-PCR]) and found no significant changes in the nasal microbiome (20). However, all tested individuals showed mild symptoms of acute upper respiratory tract infections (URTIs; such as cough, fever, and sore throat), and there was no control group of healthy individuals for comparison. Interestingly, Fan et al. analyzed COVID-19 patients’ lung biopsy specimens and found significant changes in respiratory microbiome composition following COVID-19 infection (21). This study also lacked comparison to healthy individuals.

## VOLATILE METABOLITE FORMATION DUE TO MICROBIOME ALTERATIONS

### VOCs as potential respiratory disease biomarkers.

Over the last decade, VOC analysis of air collected from the upper respiratory cavity (e.g., mouth, nose, and pharynx) for screening and early detection of respiratory diseases has been under extensive study. Lewis et al. (22) compared the volatile metabolite profiles of people diagnosed with respiratory tract infection (RTI) with breath fingerprints of people without infection by means of gas chromatography-ion mobility spectrometry (GC-IMS). This resulted in an area under the receiver operator characteristic curve (AUC-ROC) of 0.73 (95% confidence interval [CI], 0.61 to 0.86), a sensitivity of 62%, and specificity of 80% for discrimination of viral RTIs from bacterial RTIs (22). In a study of influenza, using porcine models, researchers found six VOCs (acetaldehyde, propanol, *n*-propyl acetate, methyl methacrylate, styrene, and 1,1-dipropoxy-propane) that are significantly different in the breath of infected swine compared to that of healthy controls. Another study of VOCs in the breath of influenza patients found 14 VOCs which act as biomarkers for the virus in the headspace of influenza cultures. Among those VOCs, alcohols, acids, esters, ketones, and benzene derivatives were detected. These compounds have been previously reported to be related to breath and to bacterial and viral activity in the body (23).

A new study aimed at the diagnosis of COVID-19 by means of breath analysis using GC-IMS was also recently published. Researchers were able to differentiate COVID-19-positive patients from individuals suffering other conditions (including asthma, bacterial pneumonia, and cardiac conditions) by measuring changes in 6 VOCs (ethanal, acetone, 2-butanone, monomeric methanol, dimeric methanol, and octanal) (24). They successfully discriminated between COVID-19 patients and others with >90% sensitivity. An additional study, regarding SARS-CoV-2 infections in pediatric patients, found aldehydes to be good potential COVID-19 biomarkers (especially octanal, heptanal, and nonanal), with sensitivity as high as 91% (8).

These studies demonstrate the great potential of VOCs to serve as a screening tool for diseases in general and for viral respiratory infections specifically. However, our understanding of the interplay between the pathogen itself, the host microbiome, and the host immune system remains rudimentary.

### Factors affecting the potential use of breath VOCs as a viral infection screening test.

In the process of bacterial fermentation, different classes of metabolites are formed, including VOCs (10). Thus, a change in microbiome composition and metabolism can result in a unique VOC profile, which may act as a specific biomarker for the underlying cause of change. The idea of using VOC profiles for the detection of different diseases and physiological conditions is not new (24). However, since exhaled VOCs represent different biochemical processes occurring in distinct parts of the body, the exact portion of breath collected needs to be carefully evaluated (5).

Thus, in order to enhance the signal coming from trace VOCs and to reduce any potential confounding factors (such as inhaled air composition or contaminants), there is a need to adjust the air collection methodology according to the desired target physiological process. When focusing on lung-associated VOCs (that may be generated by the lung microbiome), the last fraction of exhaled breath may be more suitable, whereas nasal VOCs would be better represented by the first part of the exhaled breath.

## CONCLUSIONS

Volatile organic compounds are products and by-products of various biological processes, those of the host as well as those from cohabitating microbial populations. Resident microbes, known collectively as the human microbiome, populate the body and connect it to the outside world at the mucosal interfaces: the gastrointestinal tract, the urogenital system, and the respiratory tract.

The microbiome can affect our susceptibility to disease, help generate an immune response to pathogens, interact with the pathogens both positively and negatively, and at the same time, be altered by different disease and physiological states.

Changes in microbiome population and function induce changes in metabolite profiles, including VOC profiles. This notion reveals the potential to detect changes in the microbiome by noninvasive methods using suitable technology. Furthermore, the underlying cause of the change may be identifiable based on specific metabolic signatures associated with specific diseases or conditions of interest. For example, one easily collected breath sample could provide clinicians with a quick diagnosis of respiratory disease such as COVID-19.

To date, studies investigating the volatilomes of COVID-19 patients indicate that there may be substantial changes in the upper and lower respiratory microbiomes due to COVID-19. Thus, we conclude that it may be possible to diagnose URTIs in general, and COVID-19 in particular, based on the VOC signature obtained from human breath. While the specificity of this method is yet to be determined, its potential as a rapid, entry-based screening tool is noteworthy. It may not be suitable for replacing molecular-based diagnostic tools (e.g., RT-PCR or antigen tests), but it can serve as a quick, easy-to-use, and modifiable first-line test.

Individual changes in volatilomes pose a significant challenge when considering the diagnostic potential of VOCs as disease biomarkers. That being said, most of the studies reviewed in this paper were cross-sectional and are notably affected by individual differences. Longitudinal studies, on the other hand, follow trends rather than specific stages. These may reduce the effect of individuality on VOC profiles, as they grant us a better understanding of the disease process and allow for comparisons of diseased and healthy states in the same individual.

To better test the hypothesis that changes in VOC patterns of COVID-19 patients result from viral-induced URT microbiome alterations, further research is needed. We suggest conducting a longitudinal study in which nasal samples are collected from healthy individuals and COVID-19 patients (both symptomatic and asymptomatic) and used for microbiome profiling via sequencing and metatranscriptomics. Functional analyses could distinguish the pathways associated with different disease states, including metabolites that serve as biomarkers (19). From these data, a target VOC signature could be identified and used in breath-based diagnostic tools with great impact.
